# Accuracy of Combined Computed Tomography Colonography and Dual Energy Iiodine Map Imaging for Detecting Colorectal masses using High-pitch Dual-source CT

**DOI:** 10.1038/s41598-018-22188-x

**Published:** 2018-02-28

**Authors:** Kai Sun, Ruijuan Han, Yang Han, Xuesen Shi, Jiang Hu, Bin lu

**Affiliations:** 1 Department of Radiology, Baotou Central Hospital, Baotou, 014040 China; 2 Department of Radiology, State Key Laboratory of Cardiovascular Disease, Fu Wai Hospital, National Center for Cardiovascular Diseases, Chinese Academy of Medical Sciences and Peking Union Medical College, Beijing, 100037 People’s Republic of China; 3 Department of Cardiology, Chaoyang Hospital, Capital Medical University, Beijing, 100020 China; 4 Department of Gastroenterology, Baotou Central Hospital, Baotou, 014040 China; 5 Department of of Surgery, Baotou Central Hospital, Baotou, 014040 China

## Abstract

To evaluate the diagnostic accuracy of combined computed tomography colonography (CTC) and dual-energy iodine map imaging for detecting colorectal masses using high-pitch dual-source CT, compared with optical colonography (OC) and histopathologic findings. Twenty-eight consecutive patients were prospectively enrolled in this study. All patients were underwent contrast-enhanced CTC acquisition using dual-energy mode and OC and pathologic examination. The size of the space-occupied mass, the CT value after contrast enhancement, and the iodine value were measured and statistically compared. The sensitivity, specificity, accuracy rate, and positive predictive and negative predictive values of dual-energy contrast-enhanced CTC were calculated and compared between conventional CTC and dual-energy iodine images. The iodine value of stool was significantly lower than the colonic neoplasia (*P* < 0.01). The sensitivity of conventional CTC was 95.6% (95% CI = 77.9%–99.2%), combined CTC and dual-energy iodine maps imaging was 95.6% (95% CI = 77.9%–99.2%). The specificity of the two methods was 42.8% (95% CI = 15.4%–93.5%) and 100% (95% CI = 47.9%–100%; *P* = 0.02), respectively. Compared with optical colonography and histopathology, combined CTC and dual-energy iodine maps imaging can distinguish stool and colonic neoplasia, distinguish between benign and malignant tumors initially and improve the diagnostic accuracy of CTC for colorectal cancer screening.

## Introduction

Computed tomography colonography (CTC) was first introduced in 1994 as a novel technique for colorectal imaging^1^ and it is increasingly disseminated as the leading radiological technique for examination of the whole colon^2,3^. CTC is an attractive alternative to optical colonoscopy because it is noninvasive and is performed without administration of analgesics or sedation, no abdominal pain during and after the procedure, no complications due obstructions, thus minimizing the risks inherent to colonoscopy. It is currently mainly in the investigation of symptomatic patients, elsewhere it is strongly advocated for colorectal cancer screening^4^. Meta-analysis data suggests CTC is effective in detecting significant colonic neoplasia, notably cancer and large polyps ≥1 cm^5^. However, standard CTC is limited to morphologic imaging and likely to be affected by remnant stool and yield false-positive diagnoses. The major diagnostic challenge of standard CTC is to differentiate stool fragments and retained fluid due to inappropriate bowel cleansing from polypoid and masses^6^. With recent advances in CT technology, dual-energy computed tomography (DECT) simultaneously acquires data sets at two different photon spectra in a single acquisition, which can be used to generate a color-coded iodine overlay image that shows the distribution of iodine within the volume of tissue^7^. The most significant contribution of DECT-based material characterization comes from the capability to assess iodine distribution through the creation of an image that exclusively shows iodine. These iodine-specific images increase tissue contrast and amplify subtle differences in attenuation between normal and abnormal tissues, improving lesion detection and characterization in the abdomen^8^. Clinical applications of DECT leverage both material-specific images and virtual monochromatic images to expand the current role of CT and overcome several limitations of standard CT^8^. Current second generation DECT with tin filter technology improves not only the dual-energy ratio difference between iodine and calcium but also the spatial resolution and CT number homogeneity of VNE images in both phantom and *in vivo* experiments^9^. Previous study^10^ has reported that using DECT, the iodine overlay and virtual nonenhanced images yields a high accuracy in staging of colorectal cancer. The aim of this study was to evaluate the diagnostic value of combined standard CTC and dual-energy iodine map imaging for detecting colorectal masses using second generation DECT, compared with optical colonography and histopathologic findings.

## Results

### Patient population

**Figure 1:**
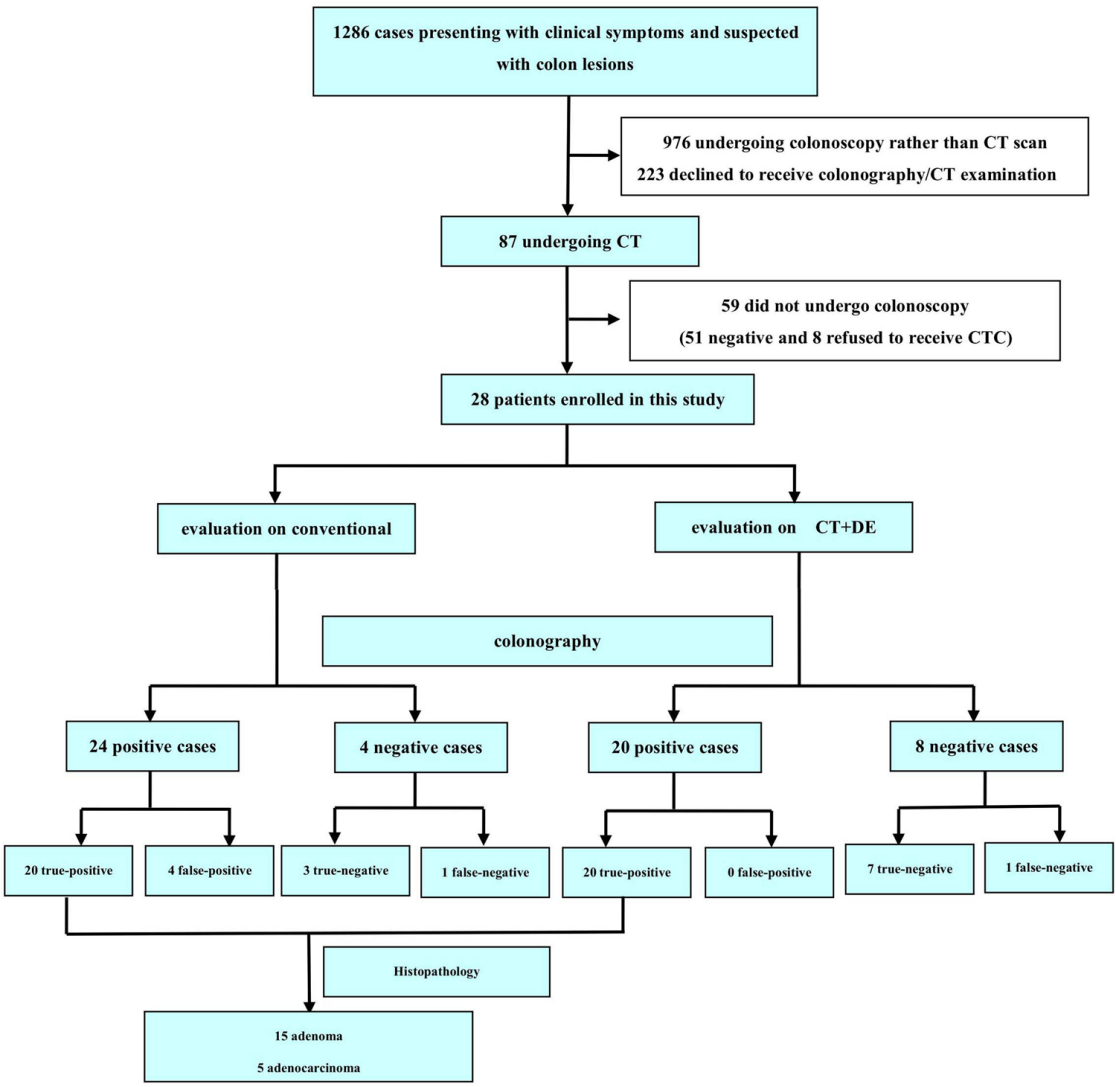
Flow chart and results of patient inclusion.

Of the 1286 patients initially identified, 1258 patients were excluded due to undergoing colonoscopy rather than CTC (976 patients), declining colonography or CTC (223 patients) and not undergoing colonoscopy (59 patients). Thus, a total of 28 patients (15 males, 13 females; 68 ± 11 years [range, 41–84 years]; average BMI, 23.8 ± 2.7 kg/m^2^ [range, 19.8~27.5 kg/m^2^]) were ultimately included. The study flowchart is illustrated in Fig. 1. No complications were noted in both CTC and OC. All patients were successfully examined by CTC, however, one patient with colon cancer failed to undergo optical colonography due to intestinal cavity stenosis. For inter-reader agreement analysis, Kappa value was 0.68 for images of CTC and DECT-based iodine maps.

### Detection rate of CTC

Twenty-four patients had a positive findings on conventional CTC but 4 of them were confirmed to be false-positive by colonography. Four patients had a negative findings on conventional CTC but one had positive finding on optical colonography and histopathology. Twenty patients were shown to be positive detected by dual-energy CT and there were no false positive results confirmed by optical colonography. Eight patients were negative on dual-energy CT, of which, one patient was a false-negative finding confirmed by optical colonography. The diameters of the the adenomas, adenocarcinomas, and stool were 1.51 ± 1.09 cm, 4.06 ± 2.82 cm, and 1.90 ± 0.748 cm, respectively. No statistical significance was observed between two groups (*P* = 0.81). The CT attentions of the adenomas, adenocarcinomas, and stool were no significantly significance. The iodine value in the adenomas, adenocarcinomas, and stool were 0.75 ± 0.11 mg/ml, 1.41 ± 0.17 mg/ml and −0.95 ± 0.44 mg/ml, respectively. The iodine value contained in the stool was significantly lower than adenomas and adenocarcinomas (*P* < 0.01). The type, size, and distribution of histopathologic findings are illustrated in Table 1.

**Table 1:** Comparison of the site, size, and qualitative measurement of colon masses by dual-energy CT.

	Adenoma (a)	Adenocarcinoma (b)	Stool (c)	*P*
Size of masses (cm)	1.51 ± 1.09	4.06 ± 2.82	1.90 ± 0.748	0.81
CT value after enhancement (Hu)	43.85 ± 7.06	52.60 ± 5.93	34.00 ± 1.41	*
Iodine value (mg/ml)	0.75 ± 0.11	1.41 ± 0.17	−0.95 ± 0.44	†
**Site**				
Rectum	3	2	1	—
Colon sigmoideum	6	2	1	—
Descending colon	9	1	0	—
Transverse colon	6	0	0	—
Colon ascendens	6	1	2	—
**Size**				
≤5 mm	11	0	1	—
6–9 mm	17	0	2	—
≥10 mm	2	6	1	—

*CT value after enhancement: comparison between a and c groups, *P* = 0.14; comparison between a and b groups, *P* = 0.09. ^†^Iodine value: comparison among a and b,c groups, *P* < 0.01.

### Histopathologic Findings

Histopathology were available for correlation in 20 positive findings detected by DECTC findings, of which 15 adenoma and 5 adenocarcinoma were confirmed in histopathologic findings.

### Diagnostic accuracy

**Figure 2:**
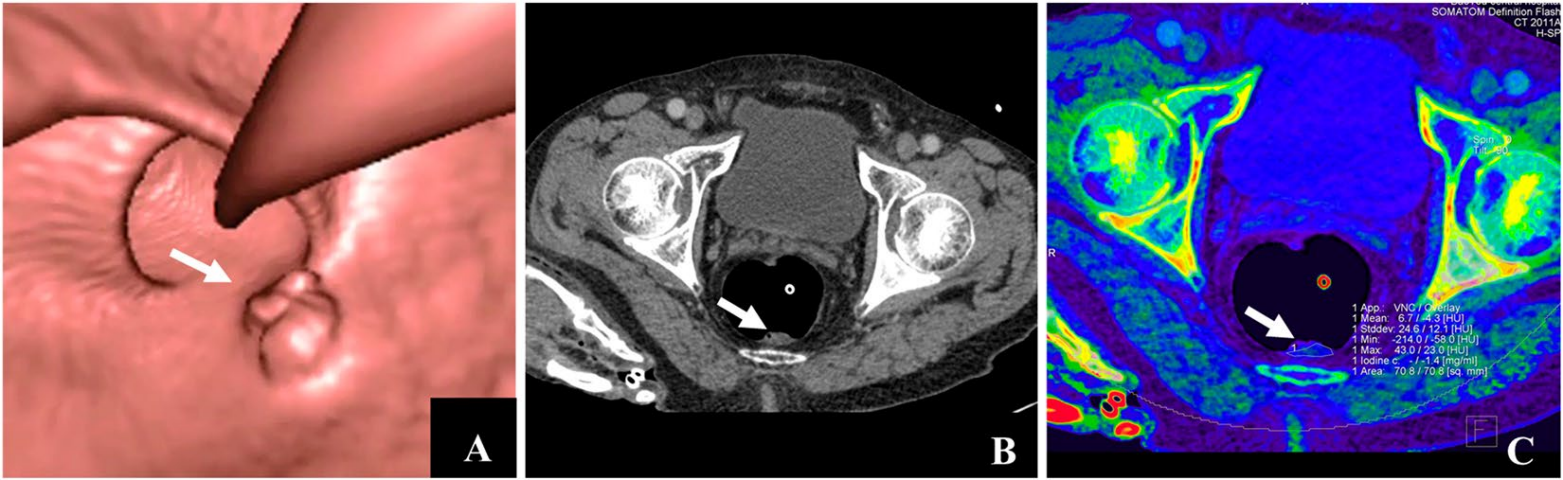
Differentiation from stool and colonic neoplasia by dual-energy CT. Male, 88 years of age. (**A,B**) denote colonography and CT axial plane image revealed irregular upheaval masses in the posterior wall of the rectum (white arrow). It is difficult to differentiate tumors from stool. (**C**) Dual-energy contrast-enhanced CT (axial plane) revealed no enhancement of masses, iodine value = −1.4 mg/ml (white arrow), suggesting no blood supply to the masses, which was validated as stool by colonography.

**Figure 3:**
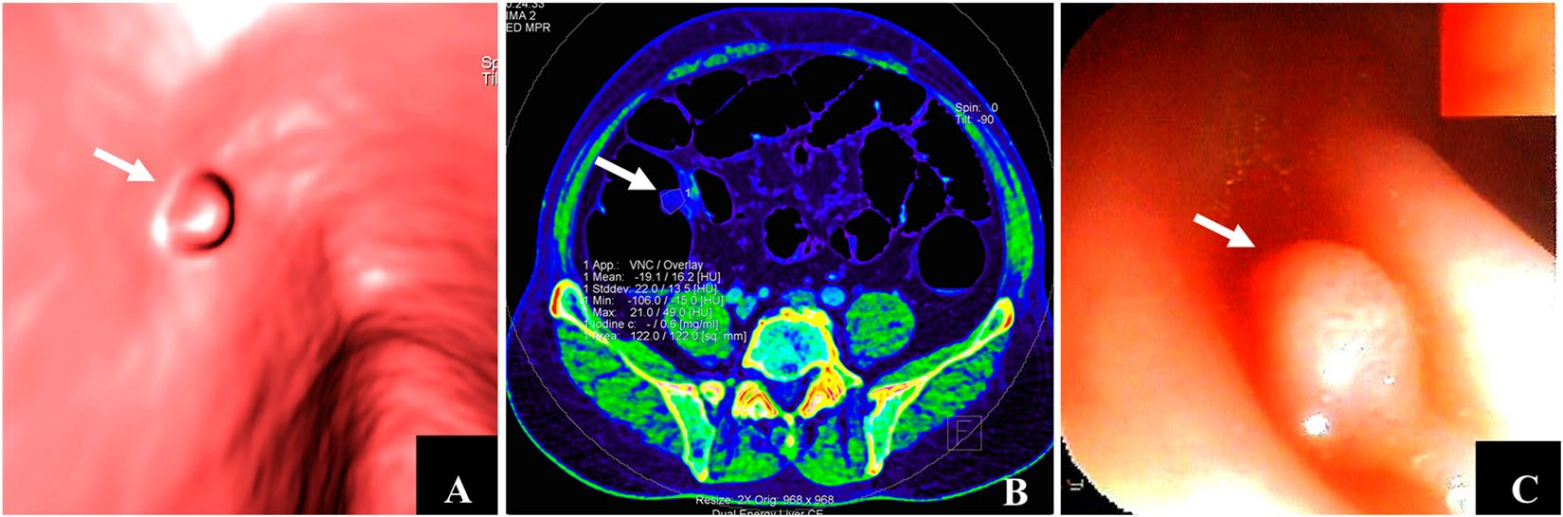
Detection of adenoma by dual-energy CT. Male, 62 years of age. (**A**) CTC revealed an 8 mm-polyp upheaval located at the colon sigmoideum with a smooth surface (white arrow). (**B**) Dual-energy contrast-enhanced CT (axial iodine image) revealed the iodine value of masses was 0.6 mg/ml (white arrow), suggesting an inadequate blood supply. (**C**) The results of adenoma were confirmed by colonography and pathologic examination (white arrow).

**Figure 4:**
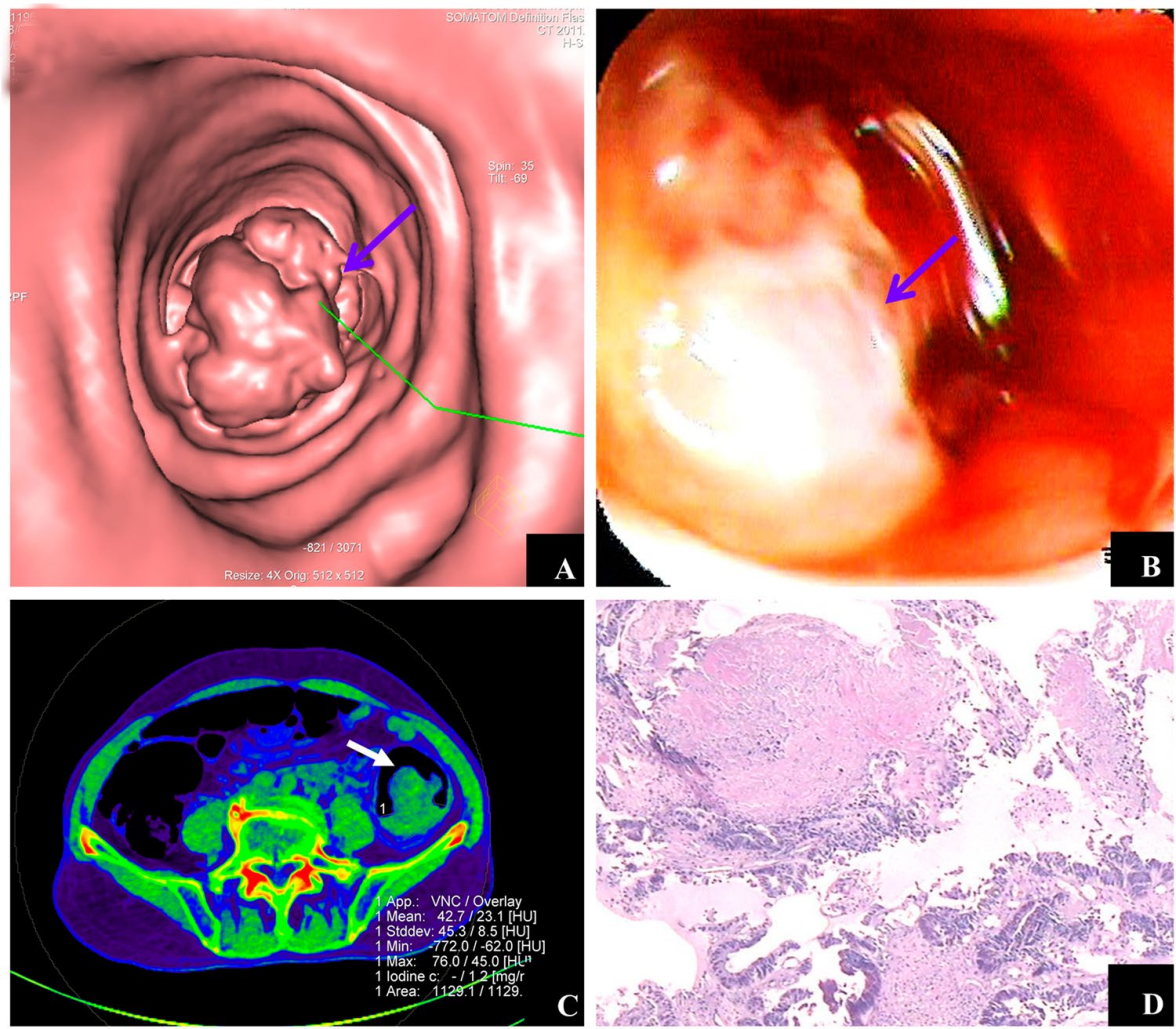
Manifestation of adenocarcinoma detected by dual-energy CT iodine image. Male, 78 years of age. (**A**) CTC revealed an irregular protruding lesion with a diameter of approximately 45.9 mm in the colon sigmoideum with a cauliflower shape (blue arrow). (**B**) Colonography revealed ulcerations, erosions, and mucous on the surface of the protruding lesion (blue arrow). (**C**) Dual-energy contrast-enhanced CT (axial iodine image) revealed the iodine value of masses was 1.2 mg/ml (white arrow), indicating sufficient blood supply of the masses. (**D**) Pathologic examination confirmed the findings of adenocarcinoma.

Conventional CTC showed masses in 4 patients, which were considered to be stools by dual-energy iodine mapping and confirmed by colonography. Compared with the pathological findings, the diagnostic accuracy of adenocarcinoma detected was significantly different between conventional CT and dual-energy CT (5.5% vs. 25%, *P* = 0.02). While the diagnostic accuracy of adenomas did not shown significant difference between the two method. The sensitivity of conventional CT and dual-energy CT in detection of masses was 95.6% (95% CI 77.9%–99.2%) and 95.6% (95% CI 77.9%–99.2%), and the specificity was 42.8% (95% CI 15.4%–93.5%) and 100% (95% CI 47.9%–100%), respectively, as shown in Table 2. Representative results are shown in Figs 2–4.

**Table 2:** Diagnostic accuracy between dual-energy and conventional CT in all t colon masses.

	Conventional CT	Dual-energy CT
True-positive	20	20
False-positive	4	0
True-negative	3	7
False-negative	1	1
Sensitivity	95.6% (95% CI 77.9%−99.2%)	95.6% (95% CI 77.9%–99.2%)
Specificity	42.8% (95% CI 15.4%– 93.5%)	100% (95% CI 47.9%–100%)
Positive predictive value (%)	83.3% (95% CI 62.6%–95.1%)	100% (95% CI 84.4%–100%)
Positive predictive value (%)	75.0% (95% CI 20.3%–95.8%)	87.5% (95% CI 36.1%–97.2%)

## Discussion

Our results demonstrate that combined CTC and dual-energy iodine map imaging allowed the detection of colorectal adenoma and adenocarcinoma with a sensitivity of 95.6% and a specificity of 100%. CTC combined with dual-energy iodine mapping may enable a reliable discrimination of enhancing colonic neoplasia (ie, adenomas and carcinomas) from benign structures (eg, stool), and it can discriminate adenoma and adenocarcinoma according to the quantify iodine uptake.

CTC is a minimally invasive imaging examination for the colon, and is safe, well tolerated and accurate for the detection of colorectal cancer (CRC) and advanced adenoma. The main challenge of CTC is the difficulty in distinguishing the remnant stool from polyps due to inadequate preparation. The patients are required to supine and prone positions during traditional CTC^11^. If the position of the masses change with the changes in posture, the masses are considered to be stool or liquid. If the positions do not change, the masses are diagnosed as polyps or solid masses^12^. Another approach is to use barium to differentiate polyps from stool^13^. However, the procedure is relatively complex and the accuracy of diagnosis is restricted.

In 2009, Karcaaltincaba *et al*.^12^ performed a first-generation dual-source CT with dual-energy mode on 8 patients with colon cancer for colonography imaging for the first time; however, the number of cases was relatively small and the field of view (FOV) of the first-generation CT scanner was limited, which did not apply to patients with large body size. A second-generation dual-source CT system installed with a spectrum photon shield (SPS) was utilized, which reduces the overlap section between high- and low-energy spectra, thereby making material separation and quantitative analysis more accurate. On the basis of the first-generation technique, the energy spectrum was purified and the FOV was expanded into 33 cm.

Dual-energy CT technology allows simultaneous acquisition of data sets at 2 different photon spectra in a single CT acquisition. Thereby, differences in material composition—ie, soft tissue polyps and contrast tagging—can be detected by variations in photon absorption at different photon energies of these actual materials and not based on differences in densities^14^. An advantage of dual-energy CT is its ability to visualize and quantify iodine uptake. Our results underline that iodine content in the case of stool remains is negative because the stool has no quantify iodine uptake^15^. Our study shows the discrimination of enhancing colonic neoplasia (ie, adenomas and carcinomas) from benign structures (eg, stool) using DECT-based iodine mapping, which will not show significant enhancement, as the similar results reported^15^ Similar results have been shown for dual-energy CT of renal masses^16^, and different enhancement of adenomas and nonadenomatous lesions has been found in MR colonography^17^. In this study,the iodine value is below zero (−0.95 ± 0.44), it maybe because there is some gas remained in the stool, which will show significant lower iodine value.

Compared with conventional CTC, the false-positive rate is significantly reduced by dual-energy CT. Boellaard *et al*.^18^ adopted dual-energy CT to diagnose colon cancer in patients without gastrointestinal tract preparation, which was consistent with the findings in the current study. The iodine value of stool was negative (−0.95 ± 0.44 mg/ml), whereas positive iodine values for adenomas and adenocarcinomas, indicating that the masses with negative iodine values were considered to be stools, differing from polyps.

Our results show that the iodine value of adenomas was significantly lower than adenocarcinomas.The iodine value of DECT-based iodine mapping contributes to differentiating adenomas from adenocarcinomas. CTC, in combination with dual-energy iodine images, can enhance the detection rate of adenomas compared with conventional CT, which is consistent with a previous report that indicated iodine images increased the sensitivity of detecting colon cancer from 90% up to 96.7%^18^. In the current study, the iodine value of different masses was quantitatively analyzed to determine the nature of the masses. Distinguishing stool from tumors and differentiating the malignancy of tumors by analyzing the blood supply of tumors are part of the innovative findings in the current study.

The results in this study revealed that the sensitivity of detecting colon masses was similar between dual-energy CT and conventional CT, while the specificity of dual-energy CT was significantly higher than traditional CT. Both the sensitivity and specificity were higher compared with 91% and 85% reported by Michael *et al*.^19^. Perry *et al*.^20^ conducted a meta-analysis and found the sensitivity was 96.1%. Margriet *et al*.^5^ conducted a meta-analysis and revealed that the detection sensitivity of adenomas with a diameter ≥6 mm were 75.9% and 82.9%, respectively, and 94.6% and 91.4% for detection specificity, respectively. The sensitivity for adenomas ≥10 mm in size were 83.3% and 87.9%, respectively, and 98.7% and 97.6% for detection specificity, respectively. Boellaard *et al*.^9^ reported that the sensitivity of dual-energy CT iodine images was 96.7%, which is similar to our findings, indicating that dual-energy contrast-enhanced CTC combined with iodine images can enhance the accuracy of identifying colon masses.

With the potential for CTC to be used as a mass-screening tool, it is important to consider radiation dose and to limit exposure wherever possible. Unlike abdominal CT, which identifies solid organ abnormalities using differences in x-ray attenuation between soft-tissue structures, CTC identifies adenoma and adenocarcinoma by exploiting the much larger attenuation differences between soft tissue and intracolonic gas, thus permitting CTC examinations to be performed at much lower radiation doses than standard CT. A survey of 34 research institutions performing CTC in 2007 evaluated the effective doses used in CTC. The mean effective dose was 5.7 mSv for screening studies and 9.1 mSv for protocols used in day-to-day routine clinical practice^21^. In our study, we evaluated the radiation doses of dual-energy CTC is 4.26 ± 1.05 mSv, which is lower than that of reported in previous studies.

This study has several limitations. First, the small patient population used in our study and the lack of a clinical following up data. Additional larger studies are warranted to fully assess the comparability of the results. Second, as most clinical scanners lack this capability of dual-energy scanning, the future universal use of this approach is questionable at this stage. Future large-scale studies are needed to more clearly establish the clinical usefulness of dual-energy contrast-enhanced CTC scanning.

## Methods

### Ethics statement

The study protocol followed the ethical guidelines of the Declaration of Helsinki and was approved by the ethics committees of BaoTou Central Hospital (Bao Tou, China). All subjects provided written informed consent and all methods were performed in accordance with the relevant guidelines and regulations.

### Research Design and Study Population

A total of 1286 patients undergoing evaluation for colon masses in our hospital between March 2012 and September 2013 were enrolled in this study. Twenty-eight patients were underwent dual-energy CTC, optical colonoscopy and pathologic examination (15 males and 13 females; age range, 41–84 years; average, 68 ± 11 years; average BMI, 23.8 ± 2.7 kg/m^2^; BMI range, 19.8~27.5 kg/m^2^). The indications for admission were as follows: irregular defecation (n = 9); positive fecal occult blood tests (n = 3); abdominal distention and pain (n = 8); weight loss (n = 5); anemia (n = 1); and other indications (n = 2). The exclusion criteria were as follows: iodine allergy; severe renal insufficiency; severe heart failure; and elderly unable to undergo examinations.

### Dual-Energy CTC Scan Protocol and image reconstruction

Three days before the examination, patients consumed a low-residue diet. One day prior to the test, the patients drank a liquid diet. The patients drank water in excess the evening before examination. At 6 a.m. on the day of the test, patients drank two portions of PEG electrolyte powder containing 13.125 g of polyethylene glycol (Shutaiqing, Staidson Beijing Biopharmaceuticals Co., Ltd., Beijing, China)^22^. Contraindications, such as glaucoma and retention of urine, were excluded. An intramuscular anisodamine injection (20 mg) was administered 10 min before the examination, to reduce intestinal motility alleviate intestinal spasms and decrease intestinal artifacts. Approximately 1000 ~ 1500 ml of gas was injected into the colon to fully expand the colon using a CB-210 remote control automatic enemator (Beijing Magnetic Wave Medical Instrument Factory, Beijing, China).

All patients were examined using a 64-section dual-source CT system (SOMATON Definition FLASH, Siemens Healthcare Sector, Forchheim, Germany) with dual-energy mode. The following acquisition parameters were used: Detector collimation, 2 × 4 × 0.6 mm; slice acquisition, 2 × 128 × 0.6 mm by means of a z-flying focal spot, with a gantry rotation time of 280 ms. The tube voltage and tube current were set as follows: pitch 1.2, tube current 104–158/91–158 mAs, and tube voltage Sn140/100 kV (140/100 kVp with tin filtering). The contrast agent (Omnipaque 350 mgI/mL; GE Healthcare, USA) was intravenously injected through the antecubital vein by a power injector (SCT-210, Medrad Incorporated, Indianola, PA, USA) with a 20-gauge needle. Contrast-agent application was controlled by the bolus-tracking technique in the ascending aorta (signal attenuation threshold, 100 HU). Data acquisition was initiated after threshold was reached in the abdominal aorta, with a mean delay of 4 seconds. A total of 60 ml of contrast agent was used, followed by 60 ml of saline solution at flow rates of 4.0 ml/s.

Image reconstruction was performed with the following parameters: a layer thickness of 1.0 mm; and an interval of 0.5 mm. Soft tissue convolution kernels (D30f) were utilized. Three-dimensional CTC post-processing images were obtained using colon software. Semi-automatic flythrough was utilized to observe pathologic changes with the intestinal cavity. Surface-shaded display (SSD) and volume-rendering (VR) images were reconstructed using three-dimensional CT scans. For the evaluation of myocardial iodine content, images from the same raw data set were then automatically reconstructed into 3 image datasets (100 kVp, 140 kVp, and an average weighted image set, M_0.3) merging 70% of the 100 kVp and 30% of the 140 kVp information). The average weighted images were used with 1.0 mm slice thickness and 0.75 mm intervals using a D30 dual energy cardiac kernel. Further post-processing was performed on a commercially available workstation (Syngo MMWP, VA36, Siemens Healthcare, Forchheim, Germany) using the dedicated dual-energy application to obtain “iodine maps” in the short axis, long axis, and 4 chamber views. Color coding was performed using shades of green (“PET” template) with bright green coding for high iodine content and black coding for a complete lack of iodine. For image analysis, the iodine maps were superimposed as a 60% overlay onto the grayscale multiplanar reformats with a slice thickness of 1.5 mm.

### Image Interpretation

All images of DECT series were evaluated independently by 2 radiologists (with 3 and 2 years of experience in DECT interpretation, respectively) who were unaware of the histopathologic findings.The site, morphology, and peripheral infiltration of the masses were recorded. The cross axle image was regarded as the standard to measure the size of masses, CT attentions after enhancement, and the amount of iodine contained on dual-energy iodine images (mg/ml). The CT and iodine values of the masses were measured. The parenchyma region was chosen as a possible area of interest. The measurement results were calculated as the mean of the measurement values from three images. Conventional CTC and dual-energy CTC with iodine images were performed to reconstruct images and derive a qualitative diagnosis of the masses.

### Estimation of radiation dose

Effective dose of CTC was calculated through the dose-length-product (DLP) multiplied by a factor coefficient (0.015). The average DLP was 283.70 ± 69.83 mGy.cm (213~446 mGy.cm).The effective radiation dose was 4.26 ± 1.05 mSv (3.20~6.69 mSv).

### Optical colonography and histopathologic findings

Optical colonoscopy was performed within 3 days. The detected masses were subject to biopsy and evaluated by two assistant directors for pathologic examination. Then, the final results of pathologic diagnosis were obtained. Matching of pathologic changes detected between CTC and optical colonography required matching of segment and size (the same or adjacent sites and the size within ±50% of colonography measurement)^23^.

### Statistical analysis

SPSS 19.0 software (SPSS, Inc., Chicago, IL, USA) was utilized for statistical analysis. Measurement data are expressed as the mean ± s. The diameter of masses, CT attentions after enhancement, and the amount of iodine were measured and statistically compared. Optical colonoscopy and pathologic examination are considered the gold standard for colonic neoplasia. The sensitivity, specificity, positive predictive value and negative predictive value of dual-energy CTC were calculated and compared with conventional CTC and dual-energy iodine images. Measurement data among different groups were compared by ANOVA. Enumeration data were statistically compared using a chi-square test. A *P* < 0.05 was considered statistically significant. Consistency among different observers was evaluated using Cohen’ Kappa analysis.
